# Heart 2 Heart: Pilot Study of a Church-Based Community Health Worker Intervention for African Americans with Hypertension

**DOI:** 10.1007/s11121-023-01553-x

**Published:** 2023-07-07

**Authors:** Elizabeth B. Lynch, Christy Tangney, Todd Ruppar, Laura Zimmermann, Joselyn Williams, LaDawne Jenkins, Steve Epting, Elizabeth Avery, Tamara Olinger, Teresa Berumen, Maggie Skoller, Rebecca Wornhoff

**Affiliations:** 1 Dept. of Family and Preventive Medicine, Rush University Medical Center, Chicago, IL USA; 2 Dept. of Clinical Nutrition, Rush University Medical Center, Chicago, IL USA; 3 Dept. of Adult Health and Gerontological Nursing, Rush University Medical Center, Chicago, IL USA; 4 Dept. of Community Health Equity and Engagement, Rush University Medical Center, Chicago, IL USA; 5 Hope Community Church, Chicago, IL USA; 6 Center for Health and Social Care Integration, Rush University Medical Center, Chicago, IL USA; 7 Dept. of Family Medicine, Boston University, Boston, MA USA

**Keywords:** African American, Black, Hypertension, Intervention, Faith-based, Health inequities, Community-based participatory research

## Abstract

African Americans (AAs) have higher prevalence of uncontrolled hypertension than Whites, which leads to reduced life expectancy. Barriers to achieving blood pressure control in AAs include mistrust of healthcare and poor adherence to medication and dietary recommendations. We conducted a pilot study of a church-based community health worker (CHW) intervention to reduce blood pressure among AAs by providing support and strategies to improve diet and medication adherence. To increase trust and cultural concordance, we hired and trained church members to serve as CHWs. AA adults (*n* = 79) with poorly controlled blood pressure were recruited from churches in a low-income, segregated neighborhood of Chicago. Participants had an average of 7.5 visits with CHWs over 6 months. Mean change in systolic blood pressure across participants was − 5 mm/Hg (*p* = 0.029). Change was greater among participants (*n* = 45) with higher baseline blood pressure (− 9.2, *p* = 0.009). Medication adherence increased at follow-up, largely due to improved timeliness of medication refills, but adherence to the DASH diet decreased slightly. Intervention fidelity was poor. Recordings of CHW visits revealed that CHWs did not adhere closely to the intervention protocol, especially with regard to assisting participants with action plans for behavior change. Participants gave the intervention high ratings for acceptability and appropriateness, and slightly lower ratings for feasibility of achieving intervention behavioral targets. Participants valued having the intervention delivered at their church and preferred a church-based intervention to an intervention conducted in a clinical setting. A church-based CHW intervention may be effective at reducing blood pressure in AAs.

In the USA, the life expectancy of African Americans (AAs) is 3.4 years shorter than that of Whites (Carnethon et al., 2017). In Chicago, the life expectancy gap between Black and non-Black residents is even larger at 9.2 years (CDPH Health Equity Index Committee, 2021). The primary cause of reduced life expectancy among AAs is cardiovascular disease (CVD) mortality, and hypertension is responsible for more CVD mortality than any other risk factor (Carnethon et al., 2017; Kochanek et al., 2013; Tsao et al., 2022). AA adults have rates of hypertension that are among the highest in the world; 58% have hypertension and only 42% have their blood pressure under control (Tsao et al., 2022). Higher rates of hypertension among AAs are due to a number of upstream factors that are rooted in systemic racism (Braveman et al., 2022), including discrimination and associated stress (Forde et al., 2020; Hicken et al., 2014; Mujahid et al., 2021), poverty, social and environmental factors that pose barriers to healthy diet and physical activity, and access to quality healthcare. These factors contribute to a higher prevalence of chronic illnesses such as hypertension (Howard et al., 2018; Thorpe Jr et al., 2008, 2014). Elimination of upstream causes is necessary to achieve social justice and health equity. However, in the short term, targeting downstream factors such as blood pressure control may be one of the most immediate ways to reduce cardiovascular mortality and improve life expectancy among AAs.

Medication and adoption of a higher quality diet are two effective strategies for reducing blood pressure (Whelton et al., 2018). Multiple studies have found that AAs are less likely to adhere to antihypertensive medications (Holmes et al., 2012; Hyre et al., 2007; Shaya et al., 2009; Siegel et al., 2007) and more likely to have lower quality diets (Ervin, 2011; Hoerr et al., 2008; Nicklas et al., 2012; Raffensperger et al., 2010; Wang et al., 2014) than other racial/ethnic groups. In multiple dietary intervention studies conducted in AA and White free-living hypertension patients, AAs showed less adherence to the recommended diet and therefore achieved less reduction in blood pressure than Whites (Epstein et al., 2012; Svetkey et al., 1999, 2005). Investigators have emphasized the need for further research into how to effectively tailor blood pressure interventions for AAs (Williams et al., 2006). Lack of adherence to medication and diet recommendations may be exacerbated for AAs because of greater mistrust of the healthcare system, another downstream consequence of systemic racism. Mistrust in the healthcare system is related to delayed blood pressure screenings for African American men (Hammond et al., 2010; Powell et al., 2019) as well as poor medication adherence for AAs with hypertension (Abel & Efird, 2013; Cuffee et al., 2013; Elder et al., 2012; Wexler et al., 2009). Interventions to improve blood pressure control in AAs may be more effective if they are delivered by a trusted interventionist in a trusted, non-clinical setting.

Community Health Workers (CHWs) have the potential to overcome trust barriers in marginalized populations (Olaniran et al., 2017). As defined by American Public Health Association (2022), CHWs are “frontline public health workers who are trusted members of, or have a close understanding of, the community served. This trusting relationship enables the worker to serve as a liaison between health/social services and the community to facilitate access to services and improve the quality and cultural competence of service delivery” (American Public Health Association, 2022). The setting of the intervention may also matter. Black churches have traditionally served as trusted service providers in AA communities and played a central role in the civil rights movement (Chaves & Tsitsos, 2001; Thomas et al., 1994). A number of lifestyle interventions have been conducted in Black churches (Hou & Cao, 2018).

A single-arm pilot study was conducted to determine the acceptability, appropriateness, feasibility, and potential effectiveness of a CHW intervention to reduce blood pressure conducted in the trusted setting of the Black church and leveraging the church social network to promote wellness among church members. CHWs were hired and trained from within a cluster of churches in a segregated, high-poverty AA neighborhood in Chicago. Blood pressure screenings were conducted at each church and participants who had at least one measure of uncontrolled blood pressure were enrolled in the study. Participants were given home blood pressure monitors and invited to meet with the CHW 12 times over 6 months to address barriers to blood pressure control. We used four of the five dimensions of the RE-AIM framework (Reach, Efficacy, Adoption and Implementation) to guide our evaluation of the potential public health benefit of the intervention.

We used the Theoretical Domains Framework (TDF), an implementation framework which guides selection of factors driving behavior change, as the conceptual framework for developing the proposed intervention (Michie et al., 2005, 2011). The TDF is centered around the COM-B system, a framework positing that behavior results from an interaction of *capability*, *opportunity*, and *motivation*. Capability is the capacity/ability of an individual to engage in the behavior, opportunity is all the factors that lie outside the individual that influence performance of the behavior, and motivation is the drive of an individual to perform the behavior. Our intervention was designed to address these three drivers of behavior. The CHW addressed *capability* by communicating educational content in a culturally concordant manner, helping participants set goals, and holding them accountable for their goals. The CHW addressed *opportunity* by providing home blood pressure monitors, addressing barriers to medication adherence and diet change, and referring participants to community resources to address needs such as food insecurity and healthcare access. The *motivation* component was addressed by using a trusted messenger to deliver the intervention within the trusted setting of the church, with support for the intervention from the pastor and other church members. The CHW was selected from the participants’ church or a similar church (in the same community with a similar denomination and culture) to increase the cultural concordance with the participant. We hypothesized that increased trust of the messenger and the intervention content and goals would lead to increased motivation to change behavior.

## Methods

### Community Engagement

This study was initiated, designed, and conducted by the Alive Faith Network (AFN), a group of researchers, pastors, and church members who are committed to working in partnership to reduce health disparities and improve the health of AAs. This partnership originated in 2009 when two African American pastors requested help from researchers (EBL) to adopt healthier lifestyles and serve as better health role models for their congregations (Lynch et al., 2019). In response, the researchers partnered with those pastors to develop a series of pastor wellness initiatives. Participation in this program promoted trust among pastors, researchers, and clinicians, and an increased commitment to healthy lifestyles on the part of pastors, for both themselves and their church members. In 2018, the AFN conducted a health screening, focused primarily on cardiometabolic risk factors and mental health, of 1106 adult church and community members in seven churches on the West Side of Chicago to prioritize church and community health needs (Lynch et al., 2020). We found that of all adults screened, 79% had elevated blood pressure (BP ≥ 120/80) at the time of the screening, 42% had blood pressure ≥ 140/90, and 50% had uncontrolled blood pressure.^1^ In discussions with pastors, it was decided that blood pressure was a high priority to address in an intervention. Pastors recommended a model akin to a parish nurse model. A parish nurse is a registered professional nurse who is a member of a faith community and addresses physical, emotional and spiritual needs of the congregation (Brudenell, 2003). As a potentially more sustainable and scalable option, we decided that we would hire and train members of participating churches to work individually with church members with hypertension to reduce their blood pressure.

### Selection and Training of Community Health Workers

An announcement was made to all participating churches that the AFN was seeking to hire and train CHWs to work on a blood pressure intervention in the churches. Recruitment and selection of CHWs were conducted in three phases. First, twelve interested individuals attended an information session led by Sinai Urban Health Institute’s Center for CHW Research, Outcomes and Workforce Development (SUHI-CROWD) which explained the role of a CHW and provided some basic information about hypertension and ways to improve blood pressure control. The two-step interview process was conducted by experienced community health workers from SUHI-CROWD and community engagement staff from Rush University Medical Center (Rush). Ten individuals participated in the first part of the interview which consisted of a role play in which the applicant played the role of a CHW when talking to an interviewer playing the role of a patient with hypertension. From this process, six individuals were selected for an interview and three individuals were hired to serve as CHWs for the program. Our initial plan was to hire one CHW from each intervention church so that CHWs would work only within their own churches. However, had we hired a CHW from each church, each CHW would have had to be part-time without benefits. In order to provide the CHWs with full-time jobs and benefits, we hired three full-time CHWs to work across seven churches.

CHWs engaged in a series of online, didactic, and experiential training activities addressing general topics in community health, patient self-management, problem-solving, social support, stress management, conflict resolution, teaching methods and strategies, system navigation, communication skills, cultural competency and humility, mental health first aid, health equity, and collaborating with medical professionals. This training was done by the lead CHW and social work staff in Rush’s community health worker hub and by SUHI-CROWD. CHWs also received training from study co-investigators, including a primary care physician, a PhD in nutrition, and a PhD in nursing, on blood pressure, hypertension, BP measurement, medication adherence, and healthy diet guidance. CHWs received over 50 h of in-person training and approximately 20 h of online training in research and healthy diet. Throughout the course of the intervention, CHWs had weekly supervision meetings with study clinicians, a clinical social worker, and the lead CHW.

### Intervention

Based on prior research (Rothschild et al., 2014), the intervention protocol specified a biweekly cadence of CHW visits, either in the home or at the church. All participants were given an Omron Evolv BP7000 Blood Pressure Monitor (Omron Healthcare, Inc. Lake Forest, IL) and taught how to correctly measure blood pressure. They were asked to self-monitor and log their BP daily. The CHWs were provided with a series of strategies to assist participants with medication adherence and diet change. Strategies addressed common barriers to antihypertensive medication adherence from the literature (Conn et al., 2015), including issues with getting refills, forgetting to take medication, and side effects. CHWs encouraged participants to see their healthcare provider if they were adherent to their medication but continued to have high blood pressure readings. Diet education modules focused on reducing sodium, increasing vegetable consumption, decreasing sugar-sweetened beverages, and increasing whole grains. Diet modules were developed with the CHWs and included interactive activities to teach participants how to read food labels to identify the amount of sodium in their foods; recognize the amount of sodium in frequently consumed foods, including fast food; integrate new vegetables into their diet; reduce sugar-sweetened beverages (SSBs) with non-sweetened choices; and replace refined foods with whole grain foods. The protocol for each visit included (1) creation of an action plan (goal) for behavior change with the participant in the form of a SMART (specific, measurable, achievable, realistic, time-bound) goal (McPherson et al., 2014), (2) review and discussion of the BP log with participants with problem-solving to identify reasons for high and low readings and translation to an action plan if needed, (3) engagement in diet module activities and translation to an action plan, and (4) review of progress on prior action plans and discussion of ways to improve/maintain behavior change using a Motivational Interviewing approach (Hettema et al., 2005). CHWs were instructed to allow the participant to guide the interaction as much as possible.

### Intervention Fidelity

For each visit, CHWs completed a visit log indicating the topics they discussed with the participants, visit length, location, etc. Approximately half of visits were recorded and 11% of recorded visits were reviewed and coded for adherence to protocol. Each of the four components of the protocol (see above) was coded on a scale from 0 to 2 where a score of 0 indicated that the step was not completed, 1 indicated it was completed but in a “rote” manner without effective problem-solving or tailoring of the information to the individual participant, and a score of 2 if the step was completed with adequate problem-solving, tailoring, and an action plan. An additional score (0–2) was given to reflect the extent to which the CHW listened to the participant and allowed the participant to direct the interaction. The scores for each visit were summed for a total possible score of 10.

### Measures

Baseline and follow-up blood pressure was measured by trained staff using an Omron HEM907XL Digital Blood Pressure Monitor (Omron Healthcare, Inc. Lake Forest, IL) after the participant sat quietly for 5 min with arm support and feet on the floor. Arm size was measured on all participants to determine appropriate cuff size. We used the average of three readings taken 2 min apart as the final measurement. Uncontrolled BP was defined as SBP ≥ 140 or SBP ≥ 130 with age ≥ 65 or self-reported cardiovascular disease, heart failure, or diabetes. Height and weight were measured using a standard portable stadiometer and a calibrated digital scale (Seca North America, Chino, CA). BMI was computed from measured height and weight. Demographic variables, medications, medical history, and healthcare access were collected via self-report. Other variables included the Confusion, Hubbub, and Order Scale (CHAOS) to measure chaos in the home environment (Matheny Jr et al., 1995), food insecurity using the Household Food Security Scale Short Form (Blumberg et al., 1999), hypertension knowledge (Sanne et al., 2008) (10-point scale, higher score indicates greater knowledge), an adapted version of the Hypertension Self-Care Profile to measure hypertension self-efficacy (Han et al., 2014) (10-item scale using a 4-point Likert scale), Beliefs in Medications Questionnaire (BMQ) (Horne & Weinman, 1999) (two 5-item scales using 5-point Likert scale, scores range from 5 to 25), and depressive symptoms (PHQ-8) (Kroenke et al., 2009). Medication adherence was measured using the Adherence to Refills and Medications Scale (ARMS) (12-items using a 4-item Likert scale), which includes two subscales: taking medications correctly (8 items) and refilling medications (4 items) (Kripalani et al., 2009). Information about medications was collected from participants at each assessment. Dietary behaviors were summarized by using a modification of the Toledo diet screener which captures the frequency of key foods and beverages critical to blood pressure control and includes a component addressing SSB consumption (Appel et al., 1997; Toledo et al., 2010). The maximum score is 8 which signifies a DASH-like pattern.

The RE-AIM dimension of *reach* was measured as the percent of individuals with uncontrolled blood pressure who participated in the study. *Efficacy* was measured as the mean pre-post change in blood pressure across study participants. *Adoption* was measured at the church level as the number of churches who participated in the study, and at the individual level as the number of individuals who had their blood pressure screened in the church, and the total number of CHW visits among study participants. *Implementation* was captured using the intervention fidelity measures described above as well as additional measures to capture participant perceptions of acceptability, appropriateness, and feasibility of the intervention (Proctor et al., 2011). Those outcomes were measured using the Acceptability of Intervention Measure (AIM), Intervention Appropriateness Measure (IAM), and Feasibility of Intervention Measure (FIM; all use a 4-point scale), which have been shown to be valid and reliable measures of those implementation constructs (Weiner et al., 2017). In addition, we included questions to capture participant perceptions of the CHW and the church-based setting (see Table 3). The study was approved by the Rush Institutional Review Board.

### Inclusion/Exclusion Criteria

Participants were eligible to participate in the study if they had at least one measure of blood pressure in the uncontrolled range, were greater than 18 years of age, were a member of a participating church, and were willing to visit with a CHW twice per month for six months. Exclusion criteria were being pregnant or breast-feeding, planning to move out of the area within the study period or on dialysis.

### Recruitment

Study coordinators were hired in each church to promote the study and encourage church members to attend the blood pressure screening. Coordinators and CHWs made announcements in church and placed information in the church bulletin during the weeks prior to the blood pressure screening. Church-wide blood pressure screenings were conducted at each church and all members were encouraged to participate. Participants with BP measures in the uncontrolled range were invited to learn about the study and complete the study screener. If they met eligibility criteria and were interested in participating, they were invited to return to the church in the subsequent week for baseline data collection, including an additional BP measurement. Participants with only one BP measurement in the uncontrolled range were considered “inconsistently controlled” and those with both BP measurements in the uncontrolled range were considered “uncontrolled.” Informed consent was obtained prior to baseline data collection.

### Statistical Analysis

Drug class of all medications was verified with a physician. Means and standard deviations are reported for continuous variables. Frequency counts and percentages are reported for categorical variables. Differences in baseline characteristics between the inconsistently controlled and uncontrolled participants were examined with two-sample *t*-tests for continuous variables and chi-square tests for categorical variables. Paired *t*-tests were used to investigate whether the change in outcome measures from baseline to follow-up was statistically different from zero at *α* = 0.05. Confidence intervals for the paired *t*-test are reported, with *p* < 0.05 if the CI did not contain zero. All analyses were performed using SAS version 9.4 (Cary, NC).

## Results

**Fig. 1:**
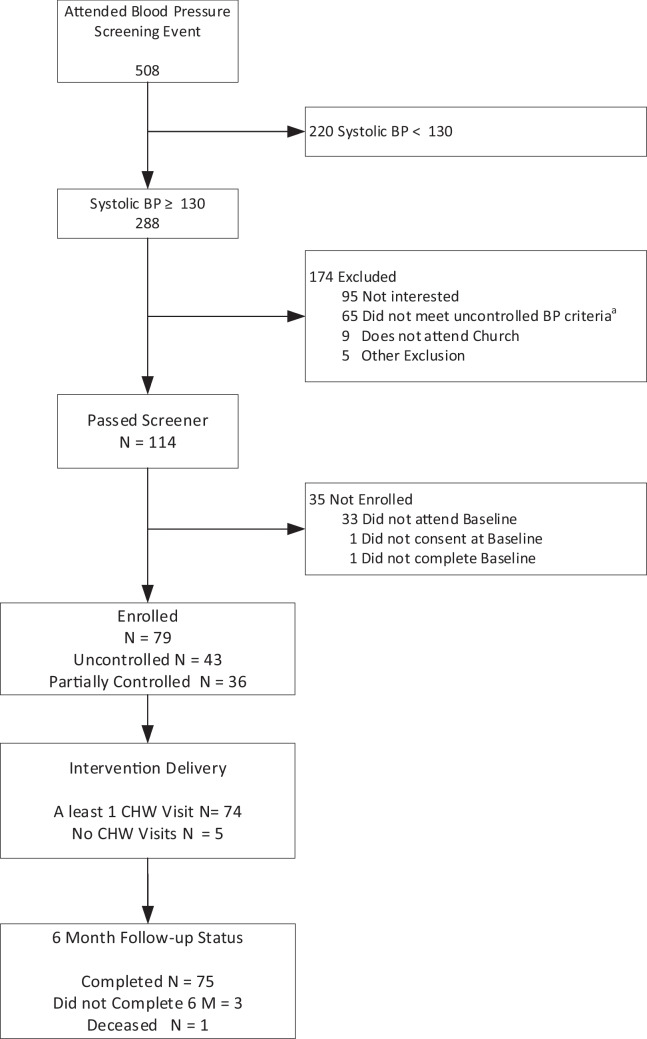
Consort diagram of participant flow through recruitment and study follow-up

Figure 1 shows the flow of participants from BP screening through study completion. Across seven churches, 508 adults were screened at the church-wide screening, 114 met eligibility criteria for the study and were interested in participating, 79 were enrolled, and 75 were included in follow-up analyses at 6 months. Tables 1 and 2 show baseline characteristics of participants. All participants were AA with a mean age of 63.4 years, 65.3% were female, and 61% had at least a high school diploma. Of the 79, 45 (57%) had uncontrolled blood pressure and the remaining 35 participants were considered to have inconsistently controlled blood pressure. Baseline BP (average of BPs for screening and baseline visit) for uncontrolled participants was 152.1 mmHg systolic (SBP) and 85.5 mmHg diastolic (DBP), and for inconsistently controlled participants, 134.1 mmHg SBP and 78.6 mmHg DBP. Many participants reported a prior diagnosis of hypertension (89.9%) and 57% reported diabetes, heart failure, or prior cardiovascular disease (CVD). Relative to participants with inconsistently controlled BP, those with uncontrolled BP were more likely to have delayed medical care due to barriers in the past year (e.g., could not get an appointment, no transportation) (31% vs 9%, *p* = 0.02), had significantly higher CHAOS scores (6.6 vs 5.5, *p* = 0.03), and were more likely to be taking no antihypertensive medications (30% vs 7%, *p* = 0.047).

**Table 1:** Baseline characteristics of study participants (total *N* = 79)

Age, mean (std)	63.4 (12.5)
Age, *N* (%)	
< 50	7 (9.7)
50–64	29 (40.3)
≥ 65	36 (50.0)
Female, *N* (%)	47 (65.3)
Live alone, *N* (%)	24 (30.4)
Education, *N* (%)	
< HS	6 (7.6)
HS	25 (31.6)
≥ HS	48 (60.8)
Employment, *N* (%)	
Employed	32 (40.5)
Unemployed	3 (3.8)
Retired	39 (49.4)
Disabled	5 (6.3)
Food Insecure, *N* (%)	12 (15.2)
No Insurance, *N* (%)	6 (7.6)
Hard to pay for the basics, *N* (%)	38 (48.1)
Self-reported hypertension, *N* (%)	71 (89.9)
Self-reported diabetes, heart failure, or prior cardiovascular disease, *N* (%)	45 (57.0)
Total chronic conditions, *N* (%)	
0	10 (12.7)
1–2	21 (26.6)
≥ 3	48 (60.8)
Delayed medical care due to barriers, *N* (%)	17 (21.5)
Did not get medical care due to financial barriers, *N* (%)	29 (36.7)
Chaos Score, means (std)	6.1 (2.2)
Mean baseline systolic BP, mean (Std)	144.4 (13.6)
Mean baseline diastolic BP, mean (Std)	82.5 (12.9)
BMI, mean (Std)	36.7 (21.1)
Obese (BMI ≥ 30), *N* (%)	48 (61)
Antihypertensive medications (*N* = 69)^a^	
Number of BP medications, *N* (%)	
0	14 (20.3)
1	21 (30.4)
2	23 (33.3)
3 +	11 (15.9)
Prescribed an ACE/ARB, *N* (%)	35 (50.7)
Prescribed a beta blocker, *N* (%)	21 (30.4)
Prescribed a diuretic, *N* (%)	22 (31.9)
Prescribed another BP Med, *N* (%)	28 (40.6)

^a^Ten participants did not have medications collected at baseline

**Table 2:** Primary and secondary outcomes at baseline, follow-up, and change

	Baseline	Follow-up	Change from baseline to follow-up	
**Primary outcome**				
**All participants**	***N* = 79**	***N* = 75**	**Mean (Std)**	**(95% CI)**^**a**^
Systolic BP (mmHg), mean (Std)	144.4 (13.6)	140.0 (18.3)	− 5.0 (19.2)	(− 9.4, − 0.5)*
Diastolic BP (mmHg), mean (Std)	82.5 (12.9)	79.3 (14.1)	− 3.6 (10.3)	(− 5.9, − 1.2)**
At least 10pt decrease systolic BP, *N* (%)	-	27 (36.0)		
**Participants with uncontrolled BP**	***N* = 45**	***N* = 42**		
Systolic BP (mmHg), mean (Std)	152.1 (11.1)	143.2 (20.9)	− 9.2 (22.1)	(− 15.9, − 2.5)**
Diastolic BP (mmHg), mean (Std)	85.5 (14.2)	80.0 (14.8)	− 5.4 (11.2)	(− 8.8, − 2.0)**
At least 10pt decrease systolic BP, *N* (%)	-	21 (47.7)		
**Participants with Inconsistently Controlled BP**	***N* = 34**	***N* = 33**		
Systolic BP (mmHg), mean (Std)	134.1 (9.2)	135.6 (12.9)	1.0 (12.2)	(− 3.4, 5.5)
Diastolic BP (mmHg), mean (Std)	78.6 (10.0)	78.4 (13.2)	− 1.0 (8.3)	(− 4.1, 2.1)
At least 10pt decrease systolic BP, *N* (%)	-	6 (19.4)		
**Secondary outcomes**	***N* = 79**	***N* = 73**		
BMI, mean (std)	36.7 (21.1)	35.2 (11.9)	− 1.5 (11.5)	(− 4.2, 1.2)
ARMS total, mean (std)	17.3 (3.4)	18.5 (5.1)	1.1 (4.5)	(0.0, 2.1)
ARMS—take medications correctly, mean (std)	9.7 (2.5)	10.1 (3.5)	0.3 (3.2)	(− 0.5, 1)
ARMS—refills, mean (std)	5.4 (1.6)	5.9 (1.8)	0.7 (1.9)	(0.2, 1.1)
Blood pressure self-efficacy, mean (std)	19.5 (5.3)	11.8 (4.1)	− 7.9 (5.7)	(− 9.2, − 6.6)***
Blood pressure knowledge, mean (std)	5.8 (1.4)	5.6 (1.5)	− 0.2 (1.7)	(− 0.6, 0.2)
Blood pressure knowledge < 70%, *N* (%)	28 (35.0)	25 (34.3)		
BMQ necessity, mean (std)	17.2 (4.9)	17.6 (5.0)	− 0.1 (4.6)	(− 1.2, 1.0)
BMQ necessity score < 15, *N* (%)	17 (21.5)	16 (22.2)		
BMQ concerns, mean (std)	12.1 (4.3)	12.8 (4.3)	0.4 (4.7)	(− 0.7, 1.5)
BMQ concerns score > 15, *N* (%)	15 (19.0)	18 (25.0)		
Toledo score (dietary intake), mean (std)	3.4 (1.2)	3.1 (0.8)	− 0.3 (1.2)	(− 0.5, 0)*
Daily servings of SSB, mean (std)	0.6 (1.1)	0.2 (0.5)	− 0.4 (0.8)	(− 0.6, − 0.2)***
Daily servings of vegetables, mean (std)	0.9 (0.7)	1.0 (0.9)	0.1 (1.1)	(− 0.1, 0.4)
Weekly servings of fast food, mean (std)	2.1 (4.1)	1.8 (3.4)	− 0.1 (2.1)	(− 0.6, 0.4)
PHQ8, mean (std)	2.5 (3.4)	2.0 (3.6)	− 0.4 (2.7)	(− 1.0, 0.2)
Depressive symptoms (PHQ8 > = 10), *N* (%)	3 (3.8)	3 (4.1)		

^**a**^Continuous variables tested using paired *t*-test, *p < 0.05 **p < 0.01 ***p < 0.001

Across 75 participants with follow-up data (95%; 3 lost and 1 deceased), change in SBP was − 5 mmHg (*p* = 0.029) and change in DBP was − 3.6 mmHg (*p* = 0.004) (see Table 2). Change in BP varied by baseline BP. Individuals with uncontrolled BP at baseline showed a change of − 9.2 mmHg SBP (*p* = 0.009) and − 5.4 mm/Hg in DBP (*p* = 0.003) whereas those with inconsistently controlled BP showed no significant changes in systolic or diastolic blood pressure. Overall, total ARMS scores (medication adherence) increased by 1.1 points (*p* = 0.046), and the increase was greater in participants who were uncontrolled at baseline (1.5-point increase, *p* = 0.076) vs. those who were inconsistently controlled at baseline (0.5 point increase, *p* = 0.38). The improvement in ARMS score appears to be primarily due to a change in the ARMS refills score for both uncontrolled and inconsistently controlled participants. BP changes (mean [std]) associated with different patterns of medication change from baseline to 6 months were as follows (66 participants had medication data at both timepoints): decreased medication (*n* = 13, − 2.2 [14.4)] mmHg), no change in medication (*n* = 26, 3.2 [14.7] mmHg), medication changed or increased (*n* = 14, − 13 [21.2] mmHg), no medications at baseline and new medications at 6 months (*n* = 5, − 17.8 [10.6] mmHg), and no medications at either timepoint (*n* = 7, − 8.1 [8.2] mmHg). Hypertension self-efficacy decreased significantly across participants. The total Toledo DASH diet score decreased but the number of daily servings of sugar-sweetened beverages (SSBs) was reduced. Participants had an average of 7.5 visits with CHWs over 6 months. The number of CHW visits was not significantly associated with blood pressure change.

### RE-AIM Measures

#### Reach

Among the individuals who participated in the blood pressure screening and met study eligibility criteria (*n* = 209), 38% enrolled in the study.

#### Adoption

All seven churches who were invited to participate in the study agreed to participate and had church members enroll in the study. An average of 72 participants were screened at each church. Though we do not have a precise number of members per church (churches did not have this number), we estimate that roughly 50% of the church members participated in the blood pressure screening per church. Almost all (94%) of the enrolled participants completed at least one CHW visit and 50.6% of participants completed at least 9 visits.

#### Implementation

Diet topics were discussed with a majority (73%) of participants. Medication adherence topics were addressed on as needed basis. The most frequently addressed topic related to medication adherence was *forgetting to take medication* (58.1% of participants), followed by *problems refilling medications* (50%), *side effects* (41.9%), *too many medications* (36.5%), and confusion about medications (28.4%).

Most visits (57.1%) lasted for 60 min or more; 89.6% of visits were completed in person, with approximately half conducted at the church (46.6%) and half conducted in the participant’s home (43%). Another adult was present at 17% of the visits. Length of visits differed by CHW, with one CHW having shorter visits than the other two (45 m vs 60 m, *p* < 0.001). BP was checked and recorded by the CHW at 99% of visits and at 83% of visits participants had logged at least one BP measure at home between visits. At 81% of visits, the CHW noted that the participant had made good or a lot of progress between visits.

Intervention fidelity was measured by coding CHW visit recordings. Recordings revealed that intervention fidelity was poor; the CHWs were frequently not following protocol. In particular, CHWs followed protocol for assisting the participant with creating action plans and discussing prior action plans in only 35% of coded visits and in 21% they did not create a new action plan or discuss a prior action plan. CHWs were more likely to follow protocol for discussion of BP logs. CHWs followed protocol for discussing BP logs in 52% of visits and met protocol for allowing participant to direct the visit at 52% of coded visits. CHWs were not expected to discuss diet at every visit. They discussed diet in 2/3 of coded visits and when they did, they followed protocol in 56% of visits.

Table 3 shows implementation outcomes for the 71 participants who completed these measures. Intervention acceptability (AIM) and appropriateness (IAM) were high, whereas feasibility (FIM) was rated slightly lower. Responses to the CHW and church-setting questions indicated satisfaction with CHWs and perceived benefit of the church setting for a blood pressure intervention.

**Table 3:** Post-intervention participant-reported implementation outcomes for H2H-CHW pilot study (*N* = 71)

	**% Agree or completely agree**	**% Completely agree**
**Acceptability of intervention measure (AIM)** (mean (SD) = 3.5 (0.5))		
The H2H program was a worthwhile use of my time	100%	45%
The Heart 2 Heart Blood pressure program met my approval	100%	45%
The Heart 2 Heart Blood pressure program appealed to me	99%	41%
I liked the Heart 2 Heart Blood pressure program	100%	47%
I welcomed the Heart 2 Heart Blood pressure program	100%	51%
**Intervention appropriateness measure (IAM)** (mean (SD) = 3.5 (0.5))		
The Heart 2 Heart Blood pressure program seems fitting for me	100%	49%
The Heart 2 Heart Blood pressure program seems suitable for me	100%	51%
The Heart 2 Heart Blood pressure program seems applicable to me	100%	46%
The Heart 2 Heart Blood pressure program seems like a good match for me	99%	46%
**Feasibility of intervention measure (FIM)** (mean (SD) = 3.2 (0.4))		
The changes I was asked to make were simple to implement.	93%	26%
The changes I was asked to make were possible	99%	29%
The changes I was asked to make were doable	99%	37%
The changes I was asked to make were easy	87%	21%
**Community health worker and program**		
My CHW helped me reduce my blood pressure.^c^	99%	38%
My CHW helped me set specific goals to improve my blood pressure.^c^	100%	50%
My CHW helped me take my medications as prescribed.^d^	86%	36%
My CHW helped me understand how to eat healthier.^b^	99%	49%
My CHW helped me realize I needed to talk to my doctor about my medications.^5^	82%	27%
My CHW was respectful of my time when scheduling and attending visits.^b^	99%	54%
I felt that my CHW cared about my health.^c^	100%	54%
I felt like my CHW cared about me as a person.^b^	100%	57%
The H2H program provided benefit to me beyond what my doctor provides.	86%	32%
I didn’t need the H2H program because I already have a doctor (or doctors).	17%	6%
**Church setting**		
I participated in the H2H program because it was in my church.^c^	96%	44%
I am more likely to participate in a health program that is based in my church than one that is based in my doctor’s office.^3^	79%	37%
The H2H Program was a good fit for my church.^b^	100%	48%
Members of my church supported me to reduce my blood pressure.^b^	91%	32%
I talked about my blood pressure with others at my church.	86%	24%
It is important that health programs are offered at church to help manage blood pressure.^b^	100%	49%
I expect that my church will provide me with resources to help control my blood pressure.	79%	27%
Church is not the appropriate setting to address health concerns like blood pressure.	10%	7%
The heart 2 heart program is a worthwhile use of church resources (time, energy, space).	100%	54%

^a^Missing 1 observation; ^b^missing 2 observations; ^c^missing 3 observations; ^d^missing 5 observations

## Discussion

This pilot intervention, conducted in churches with trained CHWs recruited from the same church community, was considered highly acceptable and appropriate to participants. Feasibility was slightly lower than acceptability and appropriateness, indicating the difficulty of behavior change to address hypertension. Nearly all participants agreed that the CHWs helped them address intervention targets and provided benefit beyond their healthcare provider. In addition, participants agreed that they participated in the program because it was in their church, that other church members supported them, and that the program was a worthwhile use of church resources. These findings suggest that conducting a CHW intervention within a church-based setting has potential to reach a population that might otherwise be unlikely to participate and that intervention effects may be enhanced by support of the church social network.

The program was effective in achieving a clinically significant reduction in blood pressure for individuals with uncontrolled blood pressure at baseline, with a mean SBP reduction of 9.2 mm/Hg and almost 50% achieving a reduction in SBP of at least 10 mmHg but had little impact for those with inconsistently controlled blood pressure. Secondary outcomes suggest that the primary mechanism of action of this intervention was increasing medication adherence and specifically, increasing timeliness of medication refills. The diet component appeared to be less effective. SSB consumption decreased, but adherence to DASH diet recommendations worsened slightly. Self-efficacy also decreased, suggesting that participants may have learned what is required to adopt a healthier diet and with experience attempting to make diet changes, realized that adhering to a heart healthy diet is more difficult than they initially anticipated. This is also consistent with somewhat attenuated feasibility ratings (relative to acceptability and appropriateness).

Coding of CHW visit recordings suggested that CHWs did not consistently follow the protocol of creating and reviewing action plans consisting of SMART goals for behavior change that were generated by participants. It is possible that greater focus on action plans would have resulted in more diet change. CHWs were more consistent at reviewing the BP logs and helping participants understand reasons for BP highs and lows. Greater focus by CHWs on blood pressure readings suggests that blood pressure changes were due to CHWs holding participants accountable for ensuring that they had BP medications available and were taking them consistently. This may be one reason why medication adherence was improved more than diet. Overall, CHWs were not highly adherent to the protocol. While the CHWs used in this study had over 50 h of training and weekly supervision from the clinical study team and an experienced CHW throughout the course of the intervention, they may have required a greater level of training, support, and monitoring in order to adhere more closely to the protocol. More research is needed on the level and type of CHW training and supervision necessary to improve the effectiveness of CHW interventions.

This study also suggests that some behavioral goals may be more appropriate for CHWs than others. In particular, the CHWs in this study seemed to be effective at increasing medication adherence, and in some cases may have helped participants acquire medications when they had none at baseline. This may be easier for the CHWs and participants because it is a concrete task with a measurable outcome (change in monitored BP). Diet change is more complex to understand, more difficult to achieve (involves more decisions and changes to lifestyle), and has fewer immediate benefits. Future research should explore the types of behavioral change that are appropriate targets for CHWs.

To date, evidence in the literature for the effectiveness of CHW interventions to improve blood pressure in AAs is weak. Of nine randomized controlled trials of CHW blood pressure interventions targeting AAs that we were able to identify from the past 20 years, only three achieved significantly greater blood pressure reductions in the intervention group relative to a control group (Becker et al., 2005; Boulware et al., 2020; Cooper et al., 2011; Hill et al., 2003; Kangovi et al., 2017; Levine et al., 2003; Lopez et al., 2017; Margolius et al., 2012; Turner et al., 2012). Each of the effective interventions included a medical professional (e.g., nurse practitioner) as an interventionist in addition to the CHW and therefore was not a true test of the capability of a CHW alone to achieve BP reduction (Becker et al., 2005; Hill et al., 2003; Turner et al., 2012). The interventions in which CHWs were the only interventionist were not effective. Use of CHWs is attractive because they can serve as trusted messengers and therefore may have greater ability to effectively communicate with patients. In addition, utilizing medical professionals as interventionists may be unsustainable given the limited resources available for clinical services offered outside and/or in addition to traditional office visits. More research is needed to maximize the potential of CHWs to reduce BP among AAs. To verify the effectiveness of the intervention described here, it must be evaluated in relation to an appropriate control group.

Most of the documented CHW intervention studies to improve BP control in AAs have recruited participants from clinical rather than community settings. Recruiting for BP interventions in community settings is challenging because it requires conducting BP screenings in the community on multiple occasions (i.e., classifying an individual as having uncontrolled BP requires measurement of BP on two separate occasions). However, the benefit of community-based interventions and recruitment is that they may help to address mistrust of healthcare that is prevalent among AAs. This study suggests that conducting a BP intervention in AA churches in partnership with pastors may increase the reach of the intervention to vulnerable populations who would not otherwise participate in a behavioral health intervention.

In conclusion, this pilot study suggested that a church-based intervention using church members trained as CHWs is feasible and acceptable to church members and may be an effective method for reducing blood pressure among AAs with uncontrolled BP. Training church members as CHWs may be a sustainable and scalable way to increase access to health information and reduce BP in the AA community.

## Data Availability

The data that support the findings of this study are available from the corresponding author, [EBL], upon reasonable request.

## Footnotes

1 Uncontrolled BP was defined as BP 140/90 or BP 130/80 with self-reported cardiovascular disease, heart failure or diabetes or systolic BP130, and age65.
