# Correction: Heart 2 Heart: Pilot Study of a Church-Based Community Health Worker Intervention for African Americans with Hypertension

**DOI:** 10.1007/s11121-024-01664-z

**Published:** 2024-03-15

**Authors:** Elizabeth B. Lynch, Christy Tangney, Todd Ruppar, Laura Zimmermann, Joselyn Williams, LaDawne Jenkins, Steve Epting, Elizabeth Avery, Tamara Olinger, Teresa Berumen, Maggie Skoller, Rebecca Wornhoff

**Affiliations:** 1 Dept. of Family and Preventive Medicine, Rush University Medical Center, Chicago, IL USA; 2 Dept. of Clinical Nutrition, Rush University Medical Center, Chicago, IL USA; 3 Dept. of Adult Health and Gerontological Nursing, Rush University Medical Center, Chicago, IL USA; 4 Dept. of Community Health Equity and Engagement, Rush University Medical Center, Chicago, IL USA; 5 Hope Community Church, Chicago, IL USA; 6 Center for Health and Social Care Integration, Rush University Medical Center, Chicago, IL USA; 7 Dept. of Family Medicine, Boston University, Boston, MA USA

**Correction to: Prevention Science** 10.1007/s11121-023-01553-x

The article “Heart 2 Heart: Pilot Study of a Church‑Based Community Health Worker Intervention for African Americans with Hypertension”, written by Lynch, E.B., Tangney, C., Ruppar, T., Zimmermann, L., Williams, J., Jenkins, L., Epting, S., Avery, E., Tamara, O., Berumen, T., Skoller, M., and Wornhoff, R., was originally published electronically on the publisher’s internet portal on 07 July 2023 without open access. With the author(s)’ decision to opt for Open Choice the copyright of the article changed on 16 February 2024 to © The Author(s) 2023 and the article is forthwith distributed under a Creative Commons Attribution 4.0 International License, which permits use, sharing, adaptation, distribution and reproduction in any medium or format, as long as you give appropriate credit to the original author(s) and the source, provide a link to the Creative Commons licence, and indicate if changes were made. The images or other third party material in this article are included in the article’s Creative Commons licence, unless indicated otherwise in a credit line to the material. If material is not included in the article’s Creative Commons licence and your intended use is not permitted by statutory regulation or exceeds the permitted use, you will need to obtain permission directly from the copyright holder. To view a copy of this licence, visit http://creativecommons.org/licenses/by/4.0/.

The original article has been corrected.

